# Serological Evidence of Foot-and-Mouth Disease Infection in Goats in Lao PDR

**DOI:** 10.3389/fvets.2020.00544

**Published:** 2020-08-20

**Authors:** Nagendrakumar B. Singanallur, Sonevilay Nampanya, Isabel MacPhillamy, Vilayvanh Soukvilay, Chattouphone Keokhamphet, Russell D. Bush, Syseng Khounsy, Navneet K. Dhand, Peter Windsor, Wilna Vosloo

**Affiliations:** ^1^Australian Centre for Disease Preparedness (Formerly Australian Animal Health Laboratory), CSIRO-Health and Biosecurity, Geelong, VIC, Australia; ^2^Sydney School of Veterinary Science, University of Sydney, Camden, NSW, Australia; ^3^National Animal Health Laboratory, Department of Livestock and Fisheries, Kounta, Vientiane, Laos

**Keywords:** transboundary animal diseases, foot and mouth disease, South East Asia, Lao PDR, small ruminants, seroprevalence

## Abstract

Foot and Mouth Disease (FMD) causes significant economic loss in Lao PDR (Laos) and perpetuates the cycle of smallholder poverty mainly through large ruminant productivity losses, increased costs of production and potential limitations to market access for trade in livestock and their products. Goats are emerging as an important livestock species in Laos, and there is an increasing trend in the number of households with goats, often farmed alongside cattle and buffalo. Although an FMD susceptible species, very little is known about the role of goats in the epidemiology of the disease in Laos. A cross-sectional seroprevalence study was conducted by detecting antibodies to the non-structural proteins (NSP), an indication of a previous infection, and serotype-specific structural proteins (SP) that could be due to vaccination or infection. The study commenced in late 2017 and sera were collected from 591 goats in 26 villages of northern, central and southern Laos. For a subset of sera samples, paired oral swab samples were also collected by a simple random sampling method to detect the prevalence of FMD virus infection at the time of collection. The NSP seroprevalence in the provinces of Borkeo and Xayabouli in the north was 42 and 8%, respectively and in Khammoune in the center, it was 20%. In the other five provinces, Luang Namtha and Luang Prabang (northern Laos), Xieng Khouang and Savannaket (central Laos), and Champasak (southern Laos), the seroprevalence was close to zero. The multivariable analysis indicated that age (*p* < 0.001) was positively associated with animal-level seropositivity and males were less likely to be seropositive than females (OR: 0.29; 95%CI: 0.10–0.83; *p* = 0.017). Continued sero-surveillance for FMD in goats is recommended to improve our understanding of their role in the epidemiology of FMD in the region and to extend support to FMD control decisions, particularly regarding vaccination.

## Introduction

Foot-and-mouth disease (FMD) is a major transboundary animal disease that is endemic in Southeast Asia, causing sporadic disease outbreaks mainly in large ruminants in the Lao People's Democratic Republic (Laos) (1, 2). The disease causes significant economic losses at both national and village levels and perpetuates the cycle of smallholder poverty through reduced animal productivity, increased cost of production, particularly from treatment costs (3, 4) and potentially, limitations to market access for trading in livestock and their products (5). The South East Asia and China FMD (SEACFMD) campaign has facilitated significant national and multilateral efforts to control FMD in the region over the past two decades (2). In partnership with SEACFMD, the Australian government funded the Stop Transboundary Animal Diseases and Zoonoses (STANDZ; 2011–2016) initiative providing important technical and financial contributions toward control of FMD in South East Asia (6). Routine FMD vaccination was a key component of the STANDZ initiative and involved the administration of 1.6 million doses of bivalent (serotype O and A) or monovalent (O) FMD vaccines to large ruminants in northern Laos between 2012 and 2016 (7). Due to the vaccine sourcing strategy conducted by the OIE vaccine banks, the vaccines are guaranteed to be high quality but may be produced by numerous different reputable manufacturers (8). The Japan Trust Fund also contributed vaccines to this program. The program targeted areas which were known to be high risk for virus transmission: areas with repeat outbreaks recorded and areas with extensive livestock trade. However, due to the lack of resources at the government level in Laos, these activities have not been continued and endemic FMD viruses (FMDV) continue to circulate in Laos, with exotic serotypes occasionally emerging (6, 9).

Goats are emerging as an important livestock species in smallholder production system in Laos with small holder livestock keepers turning away from cattle and buffalo husbandry (10) but the role of goats in the maintenance and transmission of FMD is not well-studied for this region. There is an increasing trend in the number of households with goats, often farmed alongside cattle and buffalo. Since the year 2000, the national Lao goat herd has been gradually increasing from 121,700 to 588,000 by 2017 (11). With market demands in China and Vietnam, there is increased migration of goats, along with large ruminants, through Laos in to these markets (10). Goats are rarely vaccinated for FMD in South East Asia, cattle, and buffalo are often vaccinated when donor supported official FMD vaccination programs occur in Laos (7). However, goats are occasionally vaccinated but only during an outbreak response. Several studies have shown the risk posed by FMD in small ruminants (sheep and goats) and their role in spreading the disease, acting as short-term reservoirs (12–14).

FMD has been recorded in cattle and buffaloes in Laos in the northern and central provinces between 2010 and 2017. The earliest reports in this decade were in 2010–11 in the northern provinces (1) and few outbreaks were reported in these provinces between 2013 and 2017 following widespread vaccination. The STANDZ program ceased in June 2016 and all routine vaccinations were stopped due to lack of funding. FMD outbreaks re-emerged in the northern provinces in late 2017 following cessation of the vaccination campaign in 2016. Occasional outbreaks have also been reported more recently in the central and southern provinces. A summary of the FMD outbreaks in Laos since 2011 is provided in Supplementary Table 1.

To determine the role of goats in the epidemiology of FMD in Laos, we used a cross-sectional seroprevalence study that identified antibodies to the non-structural proteins (NSP), an indication of a previous infection, and serotype-specific structural proteins (SP) that could be due to vaccination or infection. The present study aimed to estimate the seroprevalence of FMD in goats in Laos, using ten villages within the Australian Centre for International Agricultural Research (ACIAR) funded research projects on transboundary animal diseases ACIAR project AH/2012/067 (https://aciar.gov.au/project/ah-2012-067) and AH/2012/068 (https://aciar.gov.au/project/ah-2012-068) and an additional 16 non-project sites. These projects were a collaborative activity between the University of Sydney and the Department of Livestock and Fisheries, Laos and funded by the ACIAR (AH/2012/068). The projects aimed to improve smallholder livelihoods by improving transboundary animal disease risk management and enhancing biosecure beef production (ACIAR projects AH/2012/067 and AH/2012/068).

## Materials and Methods

### Study Design

**Figure 1:**
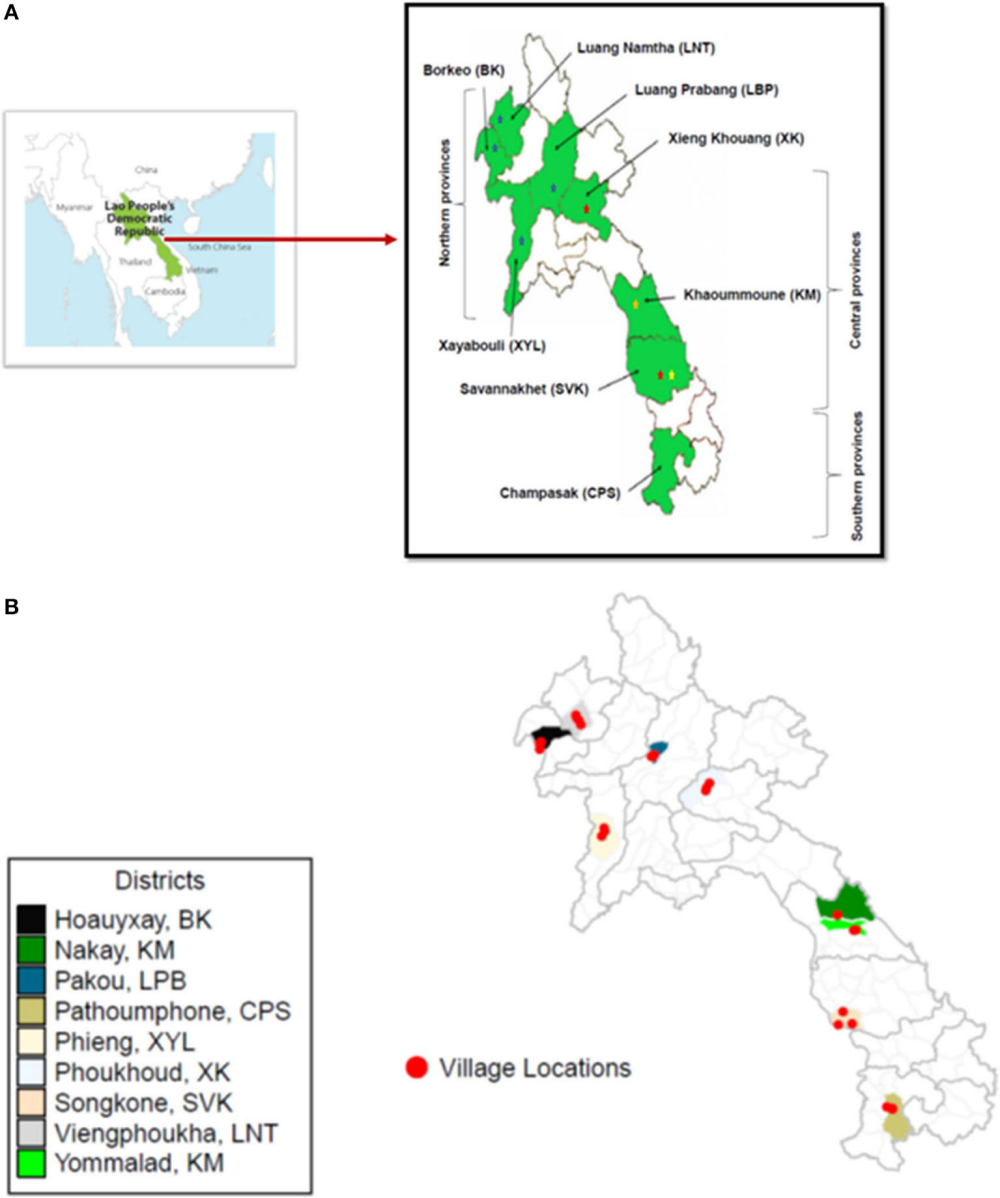
Map of Lao PDR showing provinces in the Northern, Central, and Southern regions of the country where the goat samples were collected to study the seroprevalence of FMD in goats **(A)**. The stars represent previous outbreaks recorded in cattle and buffalos in the sample areas (blue = 2010–11; red = 2014, gold = 2016, and yellow = 2017). The locations of the villages are shown in **(B)**.

Laos has seventeen provinces; each further subdivided into districts with many villages. The study was conducted between September 2017 and March 2018 in eight provinces of Laos (Figure 1; Supplementary Table 2). Five of the selected provinces were involved in FMD vaccination campaigns through the STANDZ program; in the northern and central provinces between 2012 and 2016 (7) and the central and southern provinces since 2016 funded through the New Zealand FMD control program (15). The provinces in north were Borkeo (BK), Luang Namtha (LNT), Luang Prabang (LBP), and Xayabouli (XYL); central provinces included Xieng Khouang (XK), Khoummoune (KM), and Savannakhet (SVK) and one southern Province, Champasak (CPS). Three provinces, Luang Prabang, Xieng Khouang and Xayabouli had villages actively involved in the AH/202/067 project, Savannakhet had villages actively involved in the AH/2012/068 project, and all provinces had been included at various stages in either the STANDZ or New Zealand FMD control program. The different vaccination campaigns have used either a monovalent vaccine with only an O strain (probably O1 Manisa) or a bivalent vaccine with O and A strains (probably O1 Manisa or O3039 and A Malaysia 97) sourced from a commercial vaccine manufacturer in Europe.

In 2017, there were ~588,000 goats in Laos (11). The sample size was determined with assumptions that the expected prevalence of FMD in the population was 0.005–0.01 with 95% confidence and population size >100,000 (16). In each village, 5–10 smallholder goat farmers (*n* = 134), who owned at least five goats were selected based on their willingness to participate in the survey. In each selected herd, 3–5 goats were randomly sampled (*n* = 591) resulting in a final number of 60–80 samples per province. The final study design consisted of 591 goats (445 does and 146 bucks) from 26 villages in 10 districts (Supplementary Table 2).

### Sample and Data Collection

Blood samples were collected by jugular venepuncture, using disposable syringes (5 ml) with 21G needles. In the absence of a portable centrifuge, blood was allowed to clot inside the syringes at room temperature (~30°C) with the needle on, and the separated serum was poured into serum collection tubes within 2–3 h of collection. The serum containing vials were kept in an ice bath (4–8°C) and shipped to the nearest laboratory with a freezer for long term storage at −20°C. Finally, all samples were shipped on dry ice to the National Animal Health Laboratory (NAHL), Vientiane. In each province, oral swab samples (*n* = 124) were collected by randomly choosing a goat from each household (Supplementary Table 1) using GenoTube Livestock Swabs (Thermofisher, Australia). The advantage is the samples can be shipped dry without need for a transportation medium. At least 10 oral swabs were collected from each province and stored at 4–8°C until samples were transferred to NAHL. On arrival, the swabs were transferred into lysis buffer, RNAeasy™ Mini kit (Qiagen, Germany) and stored at 4–8°C until further use.

Data were collected on animal related variables including age (in groups of <12, 12–24, and >24 months), body weight (kg) and sex (male/female) as well as grazing practices (free/forage/stall), co-grazing (yes/no); and occurrence of FMD and Orf in the last 2 years were recorded. There were no official records for vaccination of goats in any of the districts in the study area.

### Laboratory Assays

Serological assays for antibodies to the NSP and SP of FMDV were performed using Prionics kits (NS ELISA Kit and serotype O, A, and Asia1 specific cELISA kits supplied in kind by M/s. Thermofisher Scientific, Australia) at the NAHL in Vientiane. All the assays were performed as per the manufacturer's instructions, and the samples declared as positive or negative based on the per cent inhibition (PI) values (PI > 50% was positive), for the NSP and serotype specific SP assays.

Total RNA was extracted from swab samples using the RNAeasy™ Mini kit (Qiagen, Germany) following the manufacturer's instructions. The purified RNA from each oral swab sample was tested with real-time RT-PCR for detection of FMDV genome (in duplicate), using an assay targeting the IRES region (17) and the Ag-Path ID One-Step RT-PCR reagents (Applied Biosystems, Australia). Reactions were performed on the IQ-cycler CFX96 (Biorad, Australia). Samples showing a Cq>38 were considered negative. Positive and negative reaction controls were included for each plate. Ribosomal 18S RNA (18S rRNA) was used as amplification controls for the real-time RT-PCR (18).

### Statistical Analysis

Animals were classified as infected solely on the NSP result obtained; positive (1) or negative (0). The SP results were not considered for this classification due to the possibility that antibodies may be due to vaccination and not natural exposure. R 3.6.1 statistical software was used for data analysis (19); logistic regression analyses were conducted using the lme4 package (20). Univariable logistic regression was used to assess unconditional associations between potential risk factors (age, sex, weight, grazing and co-grazing practices and previous occurrence of FMD or Orf) and the outcome variable (NSP status; Positive or Negative). Grazing and co-grazing practices were coded at the farmer-level. Variables with a *p*-value <0.2 were shortlisted for the multivariable analysis. Correlations between the remaining variables were assessed using Cramer's *V* test with a cut-off of >0.30 (21). The geographic region and province/district/village level were assessed for significant difference with the Fisher's exact test.

A binomial logistic linear mixed model (LMM) was fitted for the multivariable analysis. Farmer, village, district and province were included as random effects to account for clustering, and the intraclass correlation (ICC) coefficient for each of these random terms was calculated based on the methodology described for ICC estimation from the random intercept logistic model (22). Clustering was deemed high for random effects that had an ICC greater the 0.3 (23). A backwards stepwise elimination approach was used until all variables had a *p*-value of <0.05 and were considered significantly associated with the outcome variable. Goodness-of-fit of the final regression model was assessed by calculating conditional *R*^2^ for the final model (*R*^2^_GLMM(c)_) and the amount of variation in the data explained by the fixed effects was determined by calculating marginal *R*^2^ for the fixed effects (*R*^2^_GLMM(m)_) (24). Estimated prevalence and confidence intervals were calculated using the prevalence package (v0.4.0) (25).

## Results

The details of the number of farmer households, villages, goats, mean age (±SD), and weight of goats (±SD) for each of the eight provinces from where the samples were collected are provided in Supplementary Table 2.

### Sero-Prevalence

Prevalence analysis of only the NSP antibody assay results indicated a significant difference between the provinces (*p* < 0.0005) with the highest number of positives in Borkeo (50%) and Xayabouli (12%) in the northern region, and Khammoune (27.5%) and Savannakhet (8.3%) in the central region (Figure 1, Supplementary Table 1, Table 1). Luang Prabang, Luang Namtha, Xieng Khouang, and Champasak had very low numbers of sero-positives, 0, 1.4, 1.3, and 1.3%, respectively. There was a significant difference in the seroprevalence between the three regions, i.e., north, central, and south (*p* = 0.0006), villages (*p* = 0.0005) and districts (*p* = 0.0005).

**Table 1:** Results of apparent prevalence and true prevalence of FMDV non-structural proteins antibodies from sera collected in different sample locations in northern, central, and southern Laos between September 2017 and March 2018.

**Province**	**District**	**Village**	**Samples tested**	**Number positive**	**Apparent prevalence (%) (95% CI)**
Northern Laos			300	48	16.0 (11.8–20.1)
Borkeo			76	38	50.0 (38.7–61.2)
	Hoauyxay		76	38	50.0 (38.7–61.2)
		Houaytoum	25	9	36.0 (17.2–54.8)
		Namtoy	25	13	52.0 (32.4–71.6)
		Thongseng	26	16	61.5 (42.8–80.2)
Luang Namtha			74	1	1.4 (0.0–3.9)
	Viengphoukha		74	1	1.4 (0.0–3.9)
		Khampon	16	0	0.0
		Namkieng	25	1	4.0 (0.0–11.7)
		Phadeng	23	0	0.0
		Phoulad	10	0	0.0
Luang Prabang			75	0	0.0
	Pakou		75	0	0.0
		Hadkham	25	0	0.0
		Hadkor	25	0	0.0
		Somsanouk	25	0	0.0
Xayabouli			75	9	12.0 (4.6–19.3)
	Phieng		75	9	12.0 (4.6–19.3)
		Naboum	25	9	36.0 (17.2–57.8)
		Nongheung	25	0	0.0
		Pakthang	25	0	0.0
Central Laos			216	28	12.9 (8.5–17.4)
Khoummoune			80	22	27.5 (17.7–37.3)
	Nakay		33	10	30.3 (14.6–46.0)
		Oudoumsouk	28	6	21.4 (6.2–36.6)
		Phonpadpaek	5	4	80.0 (44.9–100)
	Yommalad		47	12	25.5 (13.1–38.0)
		Nadan	28	6	21.4 (6.2–36.6)
		Phonkeo	19	6	31.6 (10.6–52.5)
Savannakhet			60	5	8.3 (1.3–15.3)
	Songkone		60	5	8.3 (1.3–15.3)
		Bengkhamlai	19	1	5.2 (0.0–15.3)
		Sabouxay	19	1	5.2 (0.0–15.3)
		Xebanghieng	22	3	13.6 (0.00–27.9)
Xieng Khouang			76	1	1.3 (0.0–3.8)
	Phoukhoud		76	1	1.3 (0.0–3.8)
		Bong	25	0	0.0
		Naxay	26	1	3.8 (0.0–11.2)
		Phouvieng	25	0	0.0
Southern Laos			75	1	1.3 (0.0–3.9)
Champasak			75	1	1.3 (0.0–3.9)
	Pathoumphone		75	1	1.3 (0.0–3.9)
		Nakok	25	0	0.0
		Nalan	25	0	0.0
		Paktouay	25	1	4.0 (0.0–11.7)
Total			591	77	13.0 (10.3–15.7)

Serotype specific ELISA kits were used to identify the proportion of animals that had antibodies to the SP of serotype O/A/Asia1 and compared that to the proportion of goats with antibodies to the NSP. A relatively low proportion of animals showed antibodies only to NSP (0–3.3%) (Table 2).

**Table 2:** Percentage of sera from goats seropositive for both FMDV non-structural proteins and structural proteins (serotype specific antibodies) collected in different provinces, districts and villages in northern, central, and southern Laos between September 2017 and March 2018.

	**Northern Laos**				**Central Laos**			**Southern Laos**
	**BK**	**LNT**	**LBP**	**XYL**	**XK**	**KM**	**SVK**	**CPS**
Total samples (*n*)	76	74	75	75	76	80	60	75
Only NSP antibodies	1.3	1.3	0	2.7	1.3	2.5	3.3	1.3
NSP and SP antibodies (%)	48.7	0	0	9.3	0	25	5	0
NSP & Serotype O only	42.1	0	0	8	0	20	3.3	0
NSP & Serotype A only	0	0	0	0	0	0	0	0
NSP & Serotype Asia1 only	0	0	0	0	0	0	0	0
NSP, Serotypes O & A	6.6	0	0	1.3	0	5.0	1.7	0
NSP, Serotypes O & Asia1	0	0	0	0	0	0	0	0
NSP, Serotypes A & Asia1	0	0	0	0	0	0	0	0
NSP, Serotypes O, A & Asia1	0	0	0	0	0	0	0	0

*BK, Borkeo; LNT, Luang Namtha; LBP, Luang Prabang; XK, Xieng Khouang; XYL, Xayabouli; KM, Khoummoune; SVK, Savannakhet; CPS, Champasak*.

Amongst the northern provinces, goats in Borkeo showed a high seroprevalence to both NSP and serotype O (42%) and NSP, serotype O and A (6.6%), while Xayabouli had 8% seroprevalence to NSP and serotype O and 1.3% to NSP and serotype O and A (Table 2). Goats in the other two northern provinces, Luang Namtha and Luang Prabang, were seronegative to all three serotypes and 1.3 and 0% seropositive to NSP.

Of the three central provinces, goats in Khammoune had the highest seroprevalence to both NSP and serotype O (20%) followed by Savannakhet (3.3%) while these two provinces also had animals that were positive for NSP antibodies along with serotype O and A (5.0 and 1.7%, respectively). Xieng Khouang in central Laos and the southern province of Champasak did not have any goats with antibodies to serotype O, A, and Asia1. Some serum samples from Luang Namtha, Xieng Khouang and Champasak were NSP antibody positive but SP antibodies negative, the PI values for the NSP results were close to the cut-off value.

Some goats did not have antibodies to NSP but were positive for antibodies only to SP (2.5–18.7%) and in some cases, to more than one serotype (Table 3). In northern Laos, the province of Xayabouli had the highest percentage of animals showing antibodies to SP of O (18.7%) with another 8% animals positive to both serotypes O and A (Table 3). Goats with antibodies to serotype O were found in all provinces. Antibodies to serotype Asia1 only were only in Champasak (1.3%) and together with serotypes O and A in Luang Namtha (1.3%) and Luang Prabang (1.3%) provinces.

**Table 3:** Percentage of goats showing serotype specific antibodies in the absence of NSP antibodies indicating exposure to FMDV vaccines from sera collected in different provinces, districts and villages in northern, central, and southern Laos between September 2017 and March 2018.

	**Northern Laos**				**Central Laos**			**Southern Laos**
	**BK**	**LNT**	**LBP**	**XYL**	**KM**	**SVK**	**XK**	**CPS**
Total samples (*n*)	76	74	75	75	80	60	76	75
Only SP antibodies (%)	2.6	12	6.7	18.7	2.5	3.4	3.9	6.7
Serotype O only (%)	2.6	4	0	6.7	2.5	1.7	3.9	2.7
Serotype A only (%)	0	0	1.8	4	0	1.7	0	2.7
Serotype Asia1 only (%)	0	0	0	0	0	0	0	1.3
Serotypes O & A (%)	0	1.3	1.3	8	0	0	0	0
Serotypes O & Asia1 (%)	0	0	0	0	0	0	0	0
Serotypes A & Asia1 (%)	0	0	0	0	0	0	0	0
Serotypes O, A & Asia1 (%)	0	6.7	4	0	0	0	0	0

*BK, Borkeo; LNT, Luang Namtha; LBP, Luang Prabang; XK, Xieng Khouang; XYL, Xayabouli; KM, Khoummoune; SVK, Savannakhet; CPS, Champasak*.

No FMDV RNA could be detected in any of the 124 oral swab samples. All oral swabs, except for one, were positive for the housekeeping gene, 18S rRNA (mean ± SD for Cp values was 27.9 ± 3.4) indicating successful extraction of total RNA and subsequent amplification of the housekeeping gene in the real-time RT-PCR (results not shown). This provides verification that the negative results are a true reflection of the virus status in the goats.

### Univariable Binomial Logistic Regression Analyses

A total of seven variables were tested for associations with FMDV serological status based on NSP antibodies, three variables at the animal-level (age, sex, and weight) and four variables at the farmer-level (grazing practices, co-grazing, occurrence of FMD, and Orf); univariable odds ratios with 95% confidence intervals (95%CI) for these are provided in Table 4. Four variables returned a *p* < 0.2 and were considered for multivariable analysis. Sabouxay village (Songkone district, Savannakhet province) was the only village containing farmers that practiced co-grazing and those who did not. The remainder of the villages had either all or no farmers practicing co-grazing.

**Table 4:** Descriptive and univariable binomial regression results for explanatory variables considered potential risk with FMDV serological status based on NSP antibodies, amongst 591 goats from 134 farmers surveyed in northern, central, and southern Laos between September 2017 and March 2018.

**Variable**	**Categories**	**FMDV NSP status**		**Total**	***p*-value^†^**	**Univariable Odds Ratio (95%CI)**
		**Negative (%)**	**Positive (%)**			
Age^§^					<0.0001	
	≤ 12 months^‡^	249 (95)	13 (5)	262		
	13–24 months	170 (83)	36 (17)	206		12.88 (4.26–39.0)
	>24 months	95 (77)	28 (23)	123		18.11 (5.72–57.35)
Sex^§^					<0.0001	
	Female^‡^	376 (84)	69 (16)	445		
	Male	138 (95)	8 (5)	146		0.16 (0.06–0.42)
Weight (kg)					<0.0001	
	≤ 15^‡^	144 (94)	9 (6)	153		
	16–30	336 (84)	66 (16)	402		11.04 (3.80–32.05)
	>30	34 (94)	2 (6)	36		5.17 (0.58–46.19)
Grazing practices					0.104	
	Free grazing^‡^	443 (89)	55 (11)	498		
	Stall Fattening	21 (66)	11 (34)	32		0.56 (0.15–2.06)
	Forage grazing	50 (82)	11 (18)	61		6.76 (1.44–31.74)
Co-grazing with large ruminants					0.858	
	No^‡^	32 (56)	25 (44)	57		
	Yes	483 (90)	52 (10)	535		0.81 (0.08–8.56)
FMD has occurred in the village and district in the last 2 years					1.00	
	No^‡^	352 (88)	50 (12)	402		
	Yes	163 (86)	27 (14)	190		1.00 (0.05–18.6)
Orf has occurred in the herd/village/district in the last 2 years					0.690	
	No^‡^	295 (95)	16 (5)	311		
	Yes	220 (78)	61 (22)	281		0.64 (0.07–5.76)

† *GLM univariable binomial logistic regression model with farmer, village, district and province included as random terms*.

§ *Variables included in the final multivariable model*.

‡ *Reference category*.

### Multivariable Mixed-Effects Logistic Regression Analyses

The final model for FMDV serological status is presented in Table 5. Only goat-level variables remained in the final model. Goat age and sex were both significantly associated with seropositivity. Older goats (>12 months of age) had higher odds of being seropositive compared to those under 12 months. Male goats had lower odds than female goats to be seropositive. The conditional *R*^2^ value for the overall model was 0.52; the marginal *R*^2^ value for the fixed effects was 0.10, indicating that the fixed effects accounted for 11.3% of the variation in the data. The variances and interclass correlation coefficients (ICCs) for the four random effect terms are shown in Table 5. The data were highly clustered at the farmer and province levels.

**Table 5:** Final multivariable mixed effects logistic regression model for FMDV serological status based on NSP antibodies, amongst 591 goats from 134 farmers surveyed in northern, central, and southern Laos between September 2017 and March 2018.

**Variables**	**β**	**SE (β)**	**OR**	**95% CI (OR)**	***p*-value**
**FIXED EFFECTS**				
Intercept	−5.42	1.13			<0.0001
Age					<0.0001
≤ 12 months	–	–	1		
13–24 months	2.30	0.56	9.97	3.32–29.89	<0.0001
>24 months	2.54	0.59	12.68	3.99–40.30	<0.0001
Sex					0.017
Female	–	–	1		
Male	−1.24	0.54	0.29	0.10–0.83	0.023

*Goodness-of-fit R_2_-test statistic $R_{\text{GLMM}\left(\text{c}\right)}^{2}$ = 0.518 $R_{\text{GLMM}\left(\text{m}\right)}^{2}$ = 0.10; Intraclass correlation coefficient: Province = 0.60; District <0.0001; Village = 0.09; Farmer = 0.42*.

## Discussion

Sound knowledge of the epidemiology of FMD in susceptible species in Laos is required to apply effective transboundary disease prevention and control measures. The epidemiology of FMD in large ruminants has been well-studied in the region (2). However, the role of small ruminants in the maintenance and transmission of FMDV in endemically infected countries has only recently received attention (13, 14, 26). Smallholder small ruminant production, particularly goats, has been emerging in Laos in recent years due to increasing regional demand, especially from China (10). However, goats are not routinely included in FMD vaccination campaigns, despite two major donor-funded FMD control programs in Laos.

This study is the first of this magnitude to report the seroprevalence of FMD in goats and the potential risk factors for FMD infection in Laos by detecting antibodies to the non-structural and structural proteins of the virus. Since goats are not routinely tested, there is a dearth of knowledge about the use of commercial serological assays for this species. In this study, commercial kits were used to determine the seroprevalence in goats and to test the application of these kits for goat sera.

The locations selected in this study have a history of routine FMD vaccination of the large ruminant populations as they are generally considered to harbor “hotspots and nodes” (areas of extensive animal trade) of FMD infection and therefore targeted to control the further spread of the disease (27). These vaccination activities likely result in a lower frequency of outbreaks, although may not necessarily stop FMDV transmission. As these are areas where FMD has historically been recognized in large ruminants, goats may be expected to have a higher seroprevalence than in those areas that have had historically had fewer outbreaks.

There is evidence in this study that some goats may have been vaccinated, with the highest proportion of animals with antibodies to the SP located in Xayabouli (18.7%) and Luang Namtha (12%). These goats had antibodies to various combinations of the serotypes in the absence of antibodies to the NSP. They could have originated from a neighboring country, where vaccination is routinely used in their national campaign for FMD control (28). It is also possible that the NSP response in these goats has decreased below detectable levels and reactions to more than one serotype could also be due to cross-reactions.

Vaccine-induced SP antibodies are only expected to remain above detectable levels for up to 6 months post-vaccination (29) and vary with the type of adjuvant (AlGel-Saponin or Oil adjuvant) used in the vaccine (30, 31). In naturally acquired FMDV infection, SP antibodies are also present. Some studies have suggested that the NSP antibodies persist for longer duration than the SP antibodies (32, 33). In fact, persistence of FMD antibodies (both SP and NSP) have been shown up to 3 years post-infection in one study (34). One study found SP antibodies to serotype A remaining at detectable level 833 days post-infection (33). Additionally, the data collection survey was unable to collect reliable life history information for the sampled animals, making it difficult to determine whether and where vaccines were administered in older animals.

The diagnostic specificity of the NSP assay used in this study is 99% in cattle (35), but the assay has not been validated for use in goats. Therefore, in the absence of clustering within a village or district, the positive samples could be the result of non-specific reactions. Another possibility of weak NSP antibody responses in some goats could be due to infection of goats leading to subclinical disease without overt clinical signs of FMD. Goats generally show mild clinical signs, and these results could indicate subclinical infection within the goat population (36).

As the provinces included in this study were purposively selected to determine if the goat population was infected in areas where outbreaks of FMD had occurred in large ruminants, selection bias was necessary, and when extrapolating the seroprevalence to the remainder of the country, caution is advised.

No antibodies to serotype Asia1 were detected in NSP positive animals, supporting the assumption that this serotype is no longer circulating in Laos (2). An outbreak in cattle was recorded in Myanmar in February 2017; the virus has been found to be closely related to samples collected in Bangladesh in 2013 (37). Although no other outbreaks caused by Asia1 have occurred prior to this study since 2006 in South East Asia (37), this serotype is still circulating in neighboring regions and with an increasingly naïve population, a reintroduction could lead to widespread outbreaks (38). Corresponding serology of the large ruminant population is required to confirm this hypothesis. Policymakers must ensure that strict biosecurity protocols are enforced to prevent the incursion of serotype Asia1 and other emerging serotypes of FMD into Laos.

Previously, free-grazing has been identified as a key risk factor for clinical FMD and NSP seropositivity in large ruminants (9). However, goats that were stall fattened were found to have a higher seroprevalence than those free-grazed (univariable OR 6.76; 95%CI 1.44–31.74). The specific differences of these practices warrant further investigation to ensure clear and consistent advice can be provided to farmers. Interestingly, in this study co-grazing was not found to be significant and goats that were co-grazed with large ruminants were marginally less likely to be seropositive (univariable OR: 0.81; 95%CI 0.08–8.56). Information was not collected on the intensity of farming for goats not co-grazed and further research is warranted to determine if there are difference in risks associated with smallholder, semi-commercial or commercial farms.

Orf outbreaks have previously been found to be incorrectly diagnosed as FMD outbreaks in Laos (10) and information on the presence of outbreaks was deemed relevant to collect. Orf outbreaks in the herd, village or district were not significantly associated with serostatus at the univariable level. However, there was a higher proportion of seropositive animals being present in an area that has had an Orf outbreak. This may warrant further investigation into factors that play a role in the spread of both diseases, and outbreak investigations are recommended to ensure the correct diagnosis is reached and appropriate control measures are implemented.

Older animals and females had higher odds of being seropositive. As females are generally retained for longer periods for breeding purposes, the likelihood they are exposed to circulating FMDV is increased, as it is for any older animal. This trend has been observed in other FMD serosurveys (14).

Cross-sectional serosurveys do not provide information regarding the temporality of disease occurrence and as a result, make it difficult to provide definitive information regarding risk factors (39). However, they do provide supporting evidence for further studies. A longitudinal serological study of proven FMD naïve animals investigating possible risk factors is recommended to determine management-related risk factors and further explore the relationship goats may play in FMDV circulation in mixed-species villages and farms. Alternatively, regular NSP antibody titer testing of targeted goat populations in recognized “hotspots” may also prove effective to further investigate the role goats play in transmission. As goats and pigs require less capital investment, they may be more likely to be present in the same villages depending on the overall socioeconomic status. It would be prudent to include pigs, goats and large ruminants in FMDV serosurveys to investigate the roles these species play in the circulation of FMDV at the village and district level in Laos (13, 14, 40). The high ICC at the province and farm level indicate the data were highly clustered with higher variance between clusters than within. This is not surprising at the farmer level as management of individual animals would be similar for each farmer and may differ between farmers. The low ICC at the village level suggests that there is a high level of variation between management practices within each village. Further investigation is warranted at the farmer and provincial level to identify any unmeasured variables that may explain the FMD serostatus compared to the animal and farm level factors explored in this study. Further, investigation of importing behaviors and goat trade movements is increasingly important for Laos (10) and is likely to provide important information that may assist provincial and national FMD control measures.

## Conclusions and Recommendations

Seroprevalence to both NSP and serotype O in Borkeo, Xayabouli and Khammoune indicate the likelihood of FMDV transmission and raising the possibility that caprine outbreaks occurred and were unrecognized. In the other provinces, the seroprevalence was close to zero, and a careful analysis of the results showed that the sera that tested NSP positive were close to the cut-off value, suggesting these may be non-specific reactions. Based on these results, and in the absence of reported clinical disease and vaccination in goats, we conclude that at least two provinces in the north and one in the center had FMDV infection in goats in the recent past. The study confirmed the utility of the NSP antibody kits and other serological kits to detect antibodies against serotype O, A and Asia1 viruses, are valuable additions for FMD sero-surveillance in this region. It should be mandatory to include goats in sero-surveillance activities for FMD in Laos and presumably other countries in the region, particularly where large scale vaccination strategies in large ruminants are planned toward FMD control and establishment of FMD free zones by vaccination in South East Asia.

## Data Availability Statement

The datasets generated for this study are available on request to the corresponding author.

## Ethics Statement

The animal study was reviewed and approved by the University of Sydney Ethics Committee (project no. 2015/765 and 2014/783, respectively) in compliance with State Acts and National Codes of Practice. Written informed consent for participation was not obtained from the owners because the samples were collected by staff of the National Animal Health Laboratory and the Department of Livestock and Fisheries, Lao PDR for their routine FMD sero-surveillance programs.

## Author Contributions

NS, SN, SK, PW, and WV contributed to the conception and design of the study. SN coordinated the field sampling. NS, VS, and CK performed the laboratory assays. IM and ND performed the statistical analysis. Funding was managed by RB, SN, SK, PW, and WV. NS wrote the first draft of the manuscript. All authors contributed to manuscript revision, read, and approved the submitted version.

## Conflict of Interest

The authors declare that the research was conducted in the absence of any commercial or financial relationships that could be construed as a potential conflict of interest.
