# Supplementary material for: Serological Evidence of Foot-and-Mouth Disease Infection in Goats in Lao PDR
Source: Front Vet Sci. 2020 Aug 20;7:544. doi: 10.3389/fvets.2020.00544 (PMC7469533; doi:10.3389/fvets.2020.00544)
Supplement: Supplementary file 1 [file Data_Sheet_1.docx]

**Supplementary Table S1: Summary of FMD outbreaks in Lao PDR recorded in cattle and buffaloes in the sample areas between 2011 and 2017.**

| Province and District | Year |
| --- | --- |
| North: Luang Namtha (Viengphoukha district)  Borkeo (Hoauyxay district)  Luang Prabang (Pakou district)  Xayabouli (Phieng district) | 2010–11 |
| North: Xieng Khouang (Bong district)  Central: Savannakhet (Songkone district) | 2014 |
| Central: Khoummoune (Nakay and Yommalad districts) | 2016 |
| Central: Savannakhet (Songkone district) | 2017 |

**Supplementary Table S2. Summary of numbers by province of participating farmers and sampled goats.**

| Province | BK | LNT | LBP | XK | XYL | KM | SK | CPS | Total |
| --- | --- | --- | --- | --- | --- | --- | --- | --- | --- |
| # of districts | 1 | 1 | 1 | 2 | 1 | 2 | 1 | 1 | 10 |
| # of villages | 3 | 4 | 3 | 3 | 3 | 4 | 3 | 3 | 26 |
| # of farmers | 18 | 29 | 18 | 16 | 18 | 24 | 17 | 19 | 159 |
| # of female goats | 53 | 60 | 51 | 50 | 55 | 64 | 45 | 68 | 446 |
| # of male goats | 23 | 15 | 24 | 26 | 20 | 16 | 15 | 7 | 146 |
| Mean ± SD age (months) | 20±14 | 23±15 | 15±6 | 18±10 | 16±10 | 17±10 | 27±17 | 16±7 | 19±12 |
| Weight ± SD (kg) | 19±6 | 17±6 | 21±6 | 24±8 | 18±6 | 20±6 | 26±8 | 19±3 | 20±7 |
| # of serum samples | 76 | 75 | 75 | 76 | 75 | 80 | 60 | 75 | 592 |
| # of swab samples | 20 | 20 | 10 | 10 | 10 | 20 | 15 | 19 | 124 |

Borkeo – BK, Luang Namtha – LNT, Luang Prabang – LBP, Xieng Khouang – XK, Xayabouli – XYL, Khoummoune – KM, Savannakhet – SVK and Champasak - CPS
